# Blocking Astrocytic GABA Restores Synaptic Plasticity in Prefrontal Cortex of Rat Model of Depression

**DOI:** 10.3390/cells9071705

**Published:** 2020-07-16

**Authors:** Ipsit Srivastava, Erika Vazquez-Juarez, Lukas Henning, Marta Gómez-Galán, Maria Lindskog

**Affiliations:** 1 Dep. Neurobiology, Care Sciences and Society, Karolinska Institutet, 171 77 Stockholm, Sweden; ipsit.srivastava@ki.se (I.S.); erika.vazquez-juarez@ki.se (E.V.-J.); Lukas.Henning@ukbonn.de (L.H.); 2 Dep. Physiology and Pharmacology, Karolinska Institutet, 171 77 Stockholm, Sweden

**Keywords:** MDD, FSL, LTP, excitation, inhibition, MAO-B, astrocyte, Deprenyl

## Abstract

A decrease in synaptic plasticity and/or a change in excitation/inhibition balance have been suggested as mechanisms underlying major depression disorder. However, given the crucial role of astrocytes in balancing synaptic function, particular attention should be given to the contribution of astrocytes in these mechanisms, especially since previous findings show that astrocytes are affected and exhibit reactive-like features in depression. Moreover, it has been shown that reactive astrocytes increase the synthesis and release of GABA, contributing significantly to tonic GABA inhibition. In this study we found decreased plasticity and increased tonic GABA inhibition in the prelimbic area in acute slices from the medial prefrontal cortex in the Flinders Sensitive Line (FSL) rat model of depression. The tonic inhibition can be reduced by either blocking astrocytic intracellular Ca^2+^ signaling or by reducing astrocytic GABA through inhibition of the synthesizing enzyme MAO-B with Selegiline. Blocking GABA synthesis also restores the impaired synaptic plasticity in the FSL prefrontal cortex, providing a new antidepressant mechanism of Selegiline.

## 1. Introduction

Despite impressive progress in our understanding of major depressive disorder (MDD) in the last decade, we still do not have a coherent picture of the etiology of the disease. It has been suggested that different brain areas related to reward, aversion, stress, etc. are implicated in the disease [1,2], with the prefrontal cortex being among those that have received special interest [3,4,5]. The importance of the prefrontal cortex is further supported by the fact that transcranial magnetic stimulation of this area is an effective treatment for depression [6].

At the neuronal level, reduced activity or an imbalance in excitation/inhibition in the prefrontal cortex has been suggested as an underlying mechanism of depression [7,8,9]. In addition, neuronal plasticity has been proven to be part of the pathology, with reduced plasticity consistently reported in depressed patients, as well as in animal models of the disease [10,11,12,13,14]. Several antidepressant treatments, including SSRIs, ketamine and endurance training have been proven to enhance plasticity [15,16,17,18,19], possibly through the common mechanism of increasing BDNF [15]. However, the interplay between the imbalance in excitation/inhibition and reduced plasticity, and their implications in depression, have not been explored in detail.

In the study of the excitation/inhibition balance, the spotlight has been directed toward neuronal activity and, in particular, to the role of phasic GABA released by inhibitory neurons [see for example [20]]. However, it is becoming evident that tonic GABA, an additional form of neuronal inhibition, is important in regulating the excitation/inhibition balance [21]. Interestingly, in pathological conditions, reactive astrocytes have been shown to contribute to this tonic inhibition through GABA synthesis in a GAD-independent pathway involving the catecholamine-oxidizing enzyme, MAO-B [22,23,24].

Astrocytes have frequently been reported to be affected in patients with depression. For example, the amount of the astrocyte-specific intermediate filament GFAP is decreased in the prefrontal cortex of depressed patients [25,26], and several astrocytic functions have been shown to be impaired in animal models of depression [27]. How these astrocyte deficiencies contribute to the pathology of depression remains to be determined, but it is well established that astrocytes play a crucial role in regulating neuronal network activity and participate in higher brain functions [28]. Thus, the role of astrocytes in the misregulation of the excitation/inhibition balance, as well as synaptic plasticity, is an underexplored target to understand and potentially treat depression.

We have previously reported that a selectively bred, well-validated animal model of depression, the FSL rat [29,30], displays reactive astrocytes and impaired plasticity in the hippocampus [11]. Here, we extend these studies to the prefrontal cortex, where we describe an impairment of long-term potentiation that can be attributed to increased astrocytic tonic inhibition. Notably, blocking the astrocytic GABA synthesizing enzyme MAO-B with Selegiline reduces tonic GABA inhibition and restores plasticity. These original findings add to other aberrant astrocytic mechanisms previously described by us in the FSL model [11,16], and further underscore the relevance of astrocytes in the pathology of depression.

## 2. Materials and Methods

All animal experiments were approved by the local ethical committee for animal research of Stockholm North in Sweden. Adult 2–3-month old male Sprague-Dawley (SD) rats obtained from Charles River (Germany) or Janvier (France), and FSL rats bred at the Karolinska Institutet, were used. All rats were group-housed in the animal facility at the Karolinska Institutet. Animals had access to food and water ad libitum, and were housed with a 12 h light/dark cycle. For the preparation of acute slices, rats were deeply anesthetized with isoflurane and decapitated soon after the disappearance of corneal reflexes.

### 2.1. Field Recordings

Brains were removed and placed in ice-cold standard aCSF. Coronal sections of the prefrontal cortex were prepared using a Leica VT1200 vibrating microtome (Leica Microsystems, Wetzlar, Germany) and placed in an interface incubation chamber filled with standard aCSF and maintained at 34 °C for 20 min, and then at room temperature for 1–2 h. Selegiline (100 μM) was added to the aCSF during the recovery period. For recording, slices were transferred to a submerged chamber with a perfusion rate of 2–3 mL per min with standard aCSF containing 130 mM NaCl, 2.4 mM NaHCO_3_, 10 mM glucose, 1.25 mM NaH_2_PO_4_, 3.5 mM KCl, 1 mM MgCl_2_ and 2 mM CaCl_2_, at 32 °C and constant bubbling with 5% CO_2_/95% O_2_.

Field excitatory postsynaptic potentials (fEPSPs) were evoked by electrical stimulation in layer two/three of the prelimbic cortex using a bipolar concentric electrode (FHC Inc., Bowdoin, ME, USA) connected to an isolated current stimulator (Digitimer, Hertfordshire, UK). The recording electrode was filled with aCSF and placed in layer five. The synaptic nature of evoked responses was tested by bath application of the AMPA receptor antagonist NBQX (25 µM). Stable fEPSP baseline responses were collected for at least 15 min using 50–60% of the maximal response. To induce LTP with reduced inhibition, we added 3 μM bicuculline; this concentration inhibits GABA_A_-mediated IPSC frequency and amplitude by 30%. LTP was induced with three trains of high-frequency stimulation (HFS) (100 pulses at 100 Hz applied at 20 s intervals). To successfully induce LTP without application of bicuculline, we used a priming tetanic stimulation (50 pulses at 100 Hz) followed 15 min later by four consecutive trains of tetanic stimulation (50 pulses at 100 Hz each, 10 s intertrain interval) [31]. Field responses were amplified 200 times, sampled at 10 kHz, low pass filtered at 2 kHz and digitally stored for offline analysis. Experiments were analyzed using the Clampfit 10.7 software (Molecular Devices, LLC, San Jose. CA, USA). For each experiment, the calculated slopes were normalized to their individual baseline periods (last 10 min) and the magnitude of LTP was determined by comparing the five-minute mean of the normalized fEPSP slope 45 min after the last train of tetanic stimulation with the mean of the last five minutes of the baseline recording.

### 2.2. Patch-Clamp Recordings

For patch clamp recordings, 300 μM coronal slices were prepared from the prefrontal cortex using a Leica VT1200 vibrating microtome (Leica Microsystems, Wetzlar, Germany) in dissection solution containing 250 mM sucrose, 2.5 mM KCl, 1.4 mM NaH_2_PO_4_, 26 mM NaHCO_3,_ 10 mM glucose, 1 mM CaCl_2_ and 4 mM MgCl_2_ (310–330 mOsm). The recovery and recording aCSF solution contained 130 mM NaCl, 3.5 mM KCl, 1.25 mM NaH_2_PO_4_, 24 mM NaHCO_3_, 10 mM glucose, 2 mM CaCl_2_ and 1.3 mM MgCl_2_. After a recovery period of a minimum of two hours, slices were transferred to a submerged recording chamber held at 32 ± 1 °C, with perfusion rate of 2–3 mL per min with standard aCSF. To block MAO-B, Selegiline (100 μM) was added to the aCSF during the recovery period.

Pyramidal neurons were identified in the layer five of the prefrontal cortex with a Carl Zeiss 40x DIC objective lens. The pClamp software (Molecular Devices, LLC, San Jose. CA, USA) was used for recording and analysis. Recordings from neurons were made using borosilicate glass pipettes with tip resistance of 4–5 MOhms filled with an intracellular solution containing 135 mM CsCl, 4 mM NaCl, 0.5 mM CaCl_2_, 10 mM HEPES, 5 mM EGTA, 2 mM Mg-ATP, 0.5 mM Na_2_-GTP and 5 mM QX-314, at a pH of 7.2–7.4, adjusted with CsOH (270–290 mOsmol). Pyramidal neurons were voltage clamped at −70 mV and access resistance was monitored throughout the recording. Recordings where access resistance changed more that 20% were rejected. GABA currents were recorded in the presence of NBQX (10 μM) and DL-AP5 (50 μM). After recording a stable baseline, picrotoxin (PTX; 100 μM) was added to block GABA_A_ receptors. Tonic GABA current was calculated as the difference in mean holding current for one minute after achieving a stable shift in baseline with PTX compared to before PTX. To correct for possible differences in soma size, all GABA currents were also compared as current densities. The tonic current density was calculated by dividing the mean tonic current by the membrane capacitance, and gave the same results as when comparing absolute currents (data not shown). Inhibitory synaptic currents (IPSCs) were identified in the same recordings before PTX administration in the Mini Analysis software (Synaptosoft, Leonia, NJ, USA), and frequency and amplitude were measured.

For calcium chelating experiments, astrocytes were identified in layer five of the prefrontal cortex by their small soma size, and confirmed by a hyperpolarized membrane potential (mean = −74.65 ± 1.32 mV, *n* = 6) and linear current voltage (IV) relationship with no action potentials. Astrocytes were patched with the Ca^2+^ chelator BAPTA in the intracellular solution containing 105 mM K-gluconate, 10 mM BAPTA, 10 mM KCl, 10 mM HEPES, 0.2 mM EGTA, 4 mM Mg-ATP, 10 mM phospohocreatine and 0.3 mM Na_2_-GTP, at a pH of 7.2–7.4, adjusted with KOH (270–290 mOsmol). AlexaFluor594 (Invitrogen, Eugene, OR, USA) was added to the intracellular solution to visualize the spread through gap junctions to neighboring astrocytes. Then, 20 min after achieving a patch in the astrocyte, an adjacent pyramidal neuron was patched and tonic currents were recorded as above. Neurobiotin was added to the neuronal patchpipette for poststaining. After the experiment, the slices were fixed in 4% paraformaldehyde and incubated for 24 h with AlexaFluor488-conjugated streptavidin. After washing and mounting, an image was taken in an epi-fluorescence microscope.

### 2.3. Immunohistochemistry

Rats were anesthetized by injecting either 80 mg/kg (FSL) or 40 mg/kg (SD) ketamine (Ketaminol, Intervet, Denmark) combined with 20 mg/kg xylazine (Xylavet, Biovet Aps, Denmark) (i.p.), and were transcardially perfused with PBS containing 4% paraformaldehyde and 0.5% glutaraldehyde. Brains were rapidly dissected and postfixed in the same fixative overnight at 4 °C. They were then cryoprotected in 0.1 M Phosphate-buffered saline (PBS) containing 30% sucrose and subsequently stored at −20 °C until cutting. First, 40-µm thick coronal sections at the level of the medial prefrontal cortex (3.20 to 2.2 mm from Bregma) were cut in a cryostat and stored at −20 °C in antifreeze solution containing 0.05 M sodium phosphate buffer, 0.44 M sucrose and 30% ethylene glycol (pH 7.6). The experimenter was blinded to the rat strain during all subsequent experiments. GFAP staining was preceded by epitope retrieval in Tris-EDTA (10 mM Tris, 1 mM EDTA, 0.05% Tween-20, pH 9.0) at 80 °C for 20 min. Sections were blocked and permeabilized using 10% normal donkey serum (NDS) and 0.3% Triton X-100 in PBS for one hour at room temperature, and subsequently incubated with primary antibodies overnight. Antibodies for S100ß (1:200, Abcam, ab4066, UK), GFAP (1:2000, Dako, Z0334, USA) and GABA (1:100, Sigma Aldrich, A2052, USA) were used. Sections were then washed and incubated with Alexa Fluor-conjugated secondary antibodies (Invitrogen, Waltham, MA, USA) in blocking solution for two hours. After washing, sections were mounted in mounting medium (Fluoromount, Sigma Aldrich, St. Louis, MO, USA).

For morphological studies, 25-µm thick z-stacks (Maximum Intensity Projections) at 1-µm intervals of combined GFAP/S100ß staining were acquired in the prelimbic area using a Nikon Eclipse Ti confocal microscope (60× objective, 16-bit, 1024 × 1024 pixels). Images were acquired at 1 airy unit (AU), and laser intensity, gain and digital offset were equally applied to all imaged samples. For GABA analyses, 8-bit images of combined S100ß/GABA immunostaining in the prelimbic regions of the prefrontal cortex were acquired using the Nikon Eclipse Ti confocal microscope (20×; 8-bit, 1024 × 1024 pixels) and a pinhole size of 1.2 AU. Images were averaged twice, and laser settings were applied as described previously. Two slices per animal were stained, and two to three images per slice were acquired.

Image analyses were performed using Fiji software (National Institute of Health, Bethesda, MD, USA) with a plug-in for Scholl analysis to assess GFAP-positive astrocyte branching. The center of mass was determined based on the S100ß-positive area. The number of GFAP-positive branches at increasing radii from the soma (5–20 µm from soma at an interval of 5 µm) was automatically determined by the software. Soma size was measured by thresholding the images with automatic triangle threshold and selecting S100ß-positive somata using the wand tracing and measurement tools implemented in the software. For GABA quantification, background fluorescence was subtracted in the GABA channel using a rolling ball radius (30 pixels), and GABA intensity was measured in regions of interest (ROI), as defined by S100ß-stained cells.

## 3. Results

### 3.1. GABA_A_ Receptor Blockage During LTP Induction Unmasks a Dysfunction of Inhibition in the Prefrontal Cortex of FSL Rats

**Figure 1:**
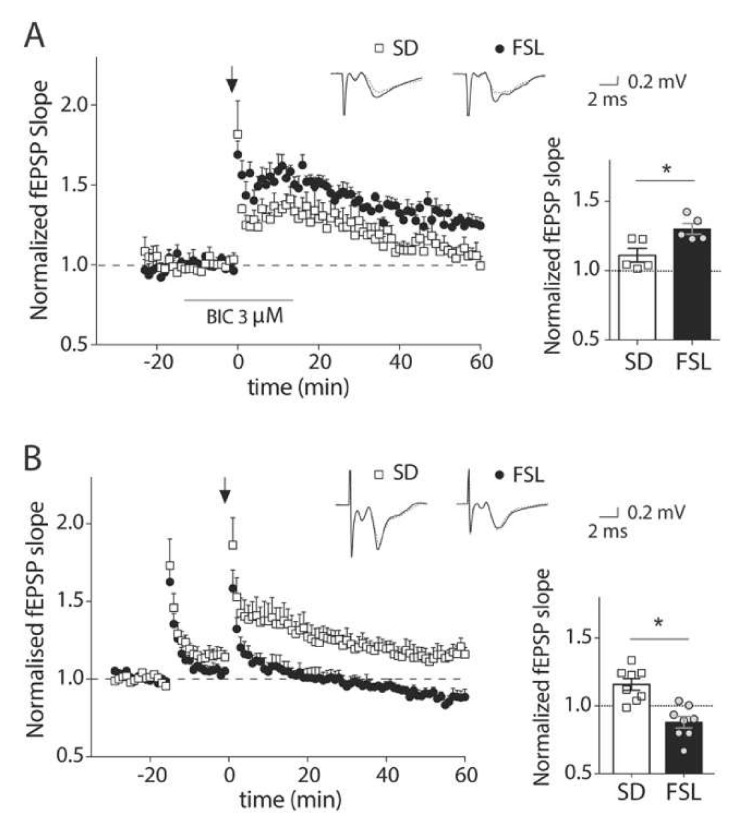
Long-term potentiation is affected in the prefrontal cortex of FSL compared to SD rats. (**A**) Timeline of mean normalized fEPSP slope evoked in the prelimbic cortex of SD and FSL rat brain slices by stimulating in layer two/three and recording from layer five, where LTP was induced with high-frequency stimulation (100 pulses at 100 Hz applied at 20 s intervals; arrow) in the presence of 3 µM bicuculline (application time indicated by line). The upper inset shows the representative averaged traces of field EPSPs before and after LTP induction (dotted and solid traces respectively). Bars (right) show mean and SEM of fEPSP slope at 40–45 min after induction with individual recordings shown as symbols revealing a significantly higher potentiation in slices of FSL rats compared to SD rats under these conditions (* *p* < 0.05, *t*-test, *n* = 5 slices/group from 3 FSL, 4 SD rats). (**B**) Timeline of mean normalized fEPSP slope evoked in the prelimbic cortex of SD and FSL rat brain slices by stimulating in layer two/three and recording from layer five, LTP was induced by delivering a priming tetanic stimulation (50 pulses at 100 Hz) followed by four trains of high-frequency stimulation (50 pulses at 100 Hz applied at 10 s intervals (arrow). The upper inset shows the representative averaged traces of field EPSPs before and after LTP induction (dotted and solid traces respectively). Bars (right) show mean and SEM of fEPSP slope at 40–45 min after induction with individual recordings shown as symbols. Long-term potentiation was observed in slices from SD rats (* = *p* < 0.05, *t*-test, *n* = 8 slices/group from 10 FSL, 5 SD rats).

Previous work in our lab has shown that a rat model of depression, the FSL rat, displays a reduced LTP amplitude in the hippocampus compared to regular Sprague Dawley (SD) rats. However, the high-frequency stimulation protocol that was optimized for the hippocampus [11] did not induce LTP in the prefrontal cortex either in SD or FSL rats (data not shown). To overcome the strong inhibition present in the prefrontal cortex that could be preventing LTP induction, we performed the LTP protocol in the presence of a subsaturating concentration of the GABA_A_ receptor antagonist, bicuculline. Surprisingly, in this condition, LTP was significantly stronger in the FSL rats compared to the SD rats (Figure 1A; mean potentiation at 40–45 min after induction 130 ± 3.9% and 111 ± 4.9% of baseline respectively, *p* < 0.05, *t*-test). This is in contrast to the decreased plasticity that we observed in the hippocampus, and raises the possibility that rather than a change in plasticity per se, the increase is due to a higher sensitivity to blocking GABA_A_ receptors. To assess this possibility, we used an alternative LTP-inducing protocol with a priming stimulation that was developed for the prefrontal cortex [31] and does not require pharmacological manipulation of the GABA system. In this condition, LTP was induced in SD (116.9 ± 5.8% of the baseline level, *p* < 0.05, *t*-test), but not in FSL rats (Figure 1B; 93.9 ± 2.9% of the baseline level), supporting the idea that the presence of stronger inhibition in the FSL rat restrains the induction of LTP in these animals. Since the priming protocol gave us the opportunity to manipulate possible targets involved in inhibition, and to evaluate its contribution to the induction of LTP, rather than inhibition itself, as in Figure 1A, this protocol was used for all the LTP experiments from Figure 1B onwards.

### 3.2. Astrocytes in the FSL Rat are Atrophic and Contain Higher Levels of GABA

**Figure 2:**
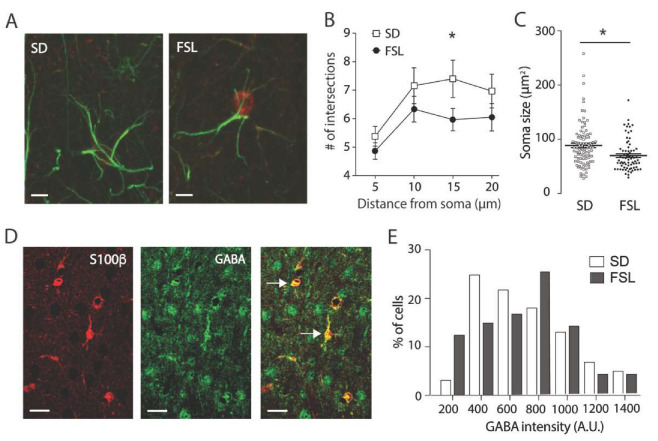
Astrocytes in the prefrontal cortex of FSL rats have reduced branching and contain GABA. (**A**) Immunostaining of prefrontal sections from SD and FSL rats with antibodies against GFAP (green) to visualize processes and S100β (red) to identify cell soma. Scale bar = 10 μm. (**B**) Scholl analysis, where the number of GFAP positive processes are counted at 5, 10, 15 and 20 μm from soma, shows that astrocytes in the FSL rat have significantly fewer branches, shown as mean number ± SEM at specific distance (Two-way ANOVA, * *p* < 0.001, *t*-test at 15 μm, * *p* < 0.01, *n* = 30 cells from four animals/group). (**C**) Soma size, as calculated as size of S100β positive areas, was significantly smaller in FSL rats compared to SD, (* *p* < 0.05 *n* = four animals/group based on average of more than 10 cells per animal). Individual cells showed in graph with mean and SEM displayed as bar and whisker. (**D**) Representative immunostaining of prefrontal section from the FSL rats with antibodies against GABA (green) and S100β (red). Scale bar = 20 μm Arrows indicate GABA positive astrocytes. (**E**) Frequency distribution of GABA staining in S100β positive cells (arbitrary units) shown as bars, illustrating the shift towards higher GABA content in astrocytes in the FSL rat (*n* = 160 cells from four animals per group, *p* < 0.005, Kolmogorov -Smirnov test).

We have previously shown increased astrocytic reactivity in the hippocampus in FSL rats, and recent data show that reactive astrocytes synthesize GABA, leading to decreased synaptic plasticity [22]. Thus, the larger LTP response in the presence of the GABA receptor blocker here observed could be due to an increased tonic GABA inhibition, arising from astrocytes that suppresses synaptic plasticity. To assess this further, we first performed morphological studies and stained astrocytes in the prefrontal cortex with antibodies against GFAP and S100β (a specific cytoplasmic and nuclear astrocytic protein present in cell bodies; Figure 2A). Astrocyte morphology was significantly different in FSL rats compared to SD, with a reduced number of GFAP positive branches (Figure 2B; Two-way ANOVA, *p* < 0.001) and smaller soma size (Figure 2C; 83 ± 2.71 μm^2^ in SD vs. 68.76 ± 5.20 μm^2^ in FSL, *p* < 0.05, *t*-test). Thus, astrocytes are affected in the prefrontal cortex but, in contrast to what we see in the hippocampus, are atrophic in the prefrontal cortex, and do not display a swollen morphology associated with reactivity. We then determined whether atrophic astrocytes also contain GABA by staining prefrontal sections with antibodies against GABA, together with S100β to identify astrocytes (Figure 2D). Mean astrocytic GABA intensity was not different in FSL rats compared to SD rats (795 vs. 785, arbitrary units); however, the median GABA intensity value was higher in the FSL group compared to SD (764 vs. 702, arbitrary units). This discrepancy between median and mean values indicates a shift in the distribution of GABA intensity per cell, with more astrocytes containing higher levels of GABA in the FSL rat. This difference in distribution is also clearly visible in the frequency histogram (Figure 2E). This discrepancy between median and mean values indicates a shift in the distribution of GABA intensity per cell, with more astrocytes containing higher levels of GABA in the FSL rat. The difference in distribution was confirmed with a Kolmogorov-Smirnov test (*p* < 0.005), and is clearly visible in the frequency histogram (Figure 2E).

### 3.3. Astrocytes Mediate Increased Tonic GABA Inhibition in FSL Rats Compared to SD

**Figure 3:**
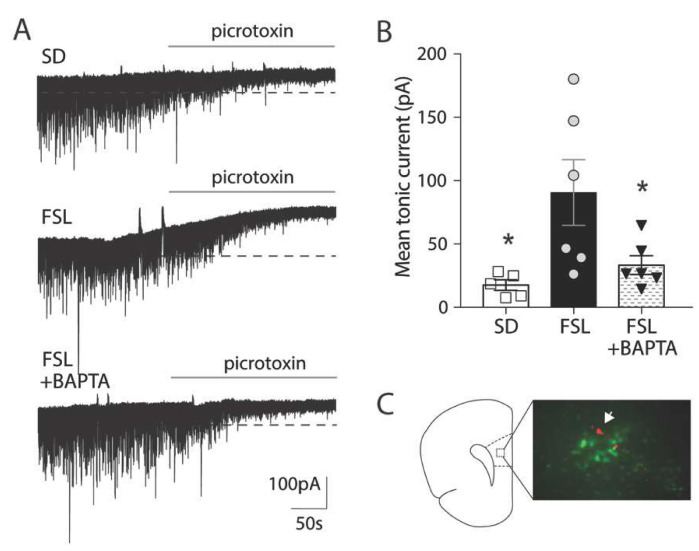
Tonic GABA current is increased in FSL compared to SD, and is mediated by astrocytes. (**A**) Representative traces showing inhibitory currents recorded from pyramidal neurons in prefrontal cortex (prelimbic area, layer five) in brain slices from 2–3-month old rats. The addition of 100 µM picrotoxin blocked synaptic currents and caused a shift of the baseline. Tonic GABA current was calculated as the amplitude of the shift before (indicated by dotted line) and after picrotoxin. (**B**) Mean tonic GABA current was significantly increased in FSL rats compared to SD rats. Filling astrocytes with the Ca^2+^ chelator BAPTA (10 mM) significantly reduced the tonic current compared to nontreated FSL slices. Bars indicate mean values, with SEM shown in error bars and the value of individual recordings shown as symbols. SD (*n* = 5 from 4 animals), FSL with BAPTA (*n* = 6 from 3 animals) and FSL rats (*n* = 6 from 5 animals) * *p* < 0.05 vs. FSL (Dunett’s multiple comparison test). (**C**) Representative micrograph illustrating the setup for dual neuronal and astrocytic patch recording with BAPTA to chelate astrocytic calcium. The astrocytic patch pipette was loaded with BAPTA together with a fluorescent dye spreading through gap junctions, allowing the visualization of the astrocytic syncytium (in green). The neuronal patch pipette contained neurobiotin that allowed for poststaining to visualize the neuron (red) and confirm the location within the targeted astrocytic syncytium.

The shift towards more cells containing higher levels of GABA in FSL rats suggests that astrocytes in the FSL rat could indeed contribute to a stronger tonic inhibition through increased release of GABA. To directly assess this effect, we recorded inhibitory currents from the layer 5 pyramidal neurons of the prefrontal cortex (prelimbic area). Tonic GABA inhibition was measured as the shift in the holding current when picrotoxin was added. Mean tonic current was indeed significantly higher in FSL rats (90.65 ± 25.92 pA) than in SD rats (Figure 3A,B; 17.63 ± 4.139 pA, *p* < 0.05, *t*-test). It has been shown that astrocytic GABA is released through Bestrophin1 channels that are activated by intracellular Ca^2+^ [23]. To confirm the astrocytic origin of the tonic inhibition, we chelated intracellular Ca^2+^ by patching an astrocyte with BAPTA in the patching pipette; this manipulation allowed BAPTA to spread to neighboring astrocytes through gap-junctions, and consequently, block calcium-sensitive GABA release from astrocytes (Figure 3C). Notably, introducing BAPTA into astrocytes induced a significant reduction of the mean tonic GABA current (33.26 ± 7.42 pA) compared to normal condition in FSL rats (Figure 3A,B; *p* < 0.05, FSL vs. FSL + BAPTA, ANOVA followed by Dunett’s multiple comparison), confirming the presence of a calcium-sensitive astrocytic origin of GABA.

### 3.4. Impaired LTP in the FSL Rat is Restored by Pretreatment with Selegiline

**Figure 4:**
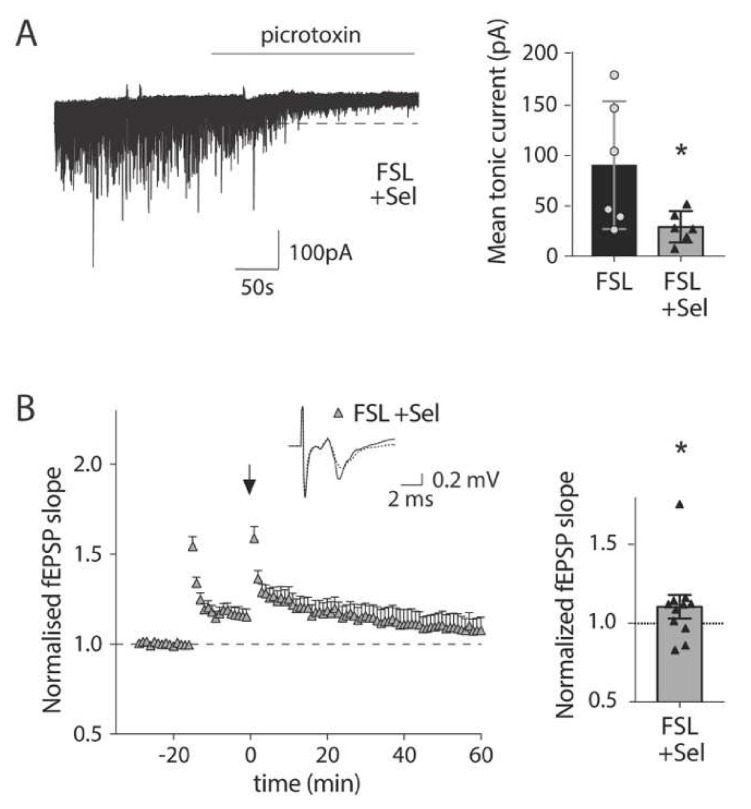
In vitro Selegiline pretreatment reduced tonic GABA and restores long-term potentiation in the prefrontal cortex of FSL rats. (**A**) Representative trace showing tonic GABA current in preincubated slices from FSL rats with Selegiline (100 µM). Selegiline preincubation of FSL slices (*n* = 6 from 4 animals) significantly reduced the mean tonic current compared to nontreated FSL slices (*n* = 6 from 5 animals, same as Figure 3). Bars indicate mean values, with SEM shown in error bars and the value of individual recordings shown as symbols (* *p* < 0.05 vs. FSL, Dunett’s multiple comparison test). (**B**) Timeline of mean normalized fEPSP slope evoked in the prelimbic cortex of FSL rat brain slices by stimulating in layer two/three and recording from layer five; LTP was induced by delivering a priming tetanic stimulation (50 pulses at 100 Hz), followed by four trains of high-frequency stimulation (50 pulses at 100 Hz applied at 10 s intervals (arrow). Bar (right) show mean and SEM of fEPSP slope 40–45 min after induction with individual recordings shown as symbols. The upper inset show the representative averaged traces of field EPSPs before and after LTP induction (dotted and solid traces respectively). Long-term potentiation was observed in slices pretreated with 100 µM Selegiline (*n* = 10 from the same ten animals as nontreated FSL slices) after 45 min (Mann Whitney test, * *p* < 0.05 vs. baseline).

In reactive astrocytes, GABA is synthesized by the enzyme monoamine oxidase B (MAO-B), a key enzyme in the putrescine degradation pathway [24] that can be blocked with Selegiline. Selegiline pre-incubation of prefrontal cortex slices from FSL rats significantly reduced the mean tonic GABA current (Figure 4A; 29.12 ± 6.35 pA with Selegiline vs. 90.65 ± 25.92 pA in nontreated slices, *p* < 0.05, FSL vs. FSL + Selegiline, ANOVA followed by Dunett’s multiple comparison. Note that data from nontreated slices is the same as above). Neuronally released GABA, measured as an increase in the frequency of inhibitory postsynaptic currents, was higher in FSL rats compared to SD rats, but was not affected by Selegiline (Supp. Figure S1). Finally, to confirm the contribution of the increased astrocytic tonic inhibition in the LTP suppression observed in FSL rats (Figure 1B), LTP was again evaluated in slices from the FSL rat that had been pretreated with Selegiline. Indeed, under these conditions, LTP was restored in the FSL rat (Figure 4B; 110.5% ± 7.4% of the baseline level, *p* < 0.05, *t*-test).

## 4. Discussion

Here, we show that astrocytes in the prefrontal cortex of the FSL rat display an atrophic morphology with reduction in branching and soma size. This is consistent with what has been shown in depressed patients [25,26], where reduced GFAP staining has been observed. Moreover, we show that astrocytes in the prefrontal cortex in an animal model of depression contain more GABA, that contributes to an increased tonic inhibition of pyramidal cells in the area (Figure 2 and Figure 3). Such an increase has previously only been described in animal models of Alzheimer’s disease [22], where it was associated with a swollen morphology of astrocytes. Moreover, we suggest that the increase in astrocytic GABA has a profound effect on synaptic plasticity in the prefrontal cortex (Figure 1 and Figure 4).

GABA in the extracellular space can activate extrasynaptic GABA_A_ receptors and cause tonic inhibition of neurons [21]. In the dentate gyrus of the hippocampus, tonic inhibition has been shown to contribute to an inhibitory modulation of LTP [32]. Moreover, reactive astrocytes in an animal model of Alzheimer’s disease have been shown to synthesize and release GABA, thus increasing tonic inhibition [33,34] and affecting the excitation/inhibition balance, as well as impairing LTP [22,35]. Our data from the prefrontal cortex in an animal model of depression is consistent with these results, and suggests that increased tonic inhibition and its impact on synaptic plasticity may be a general feature of dysfunctional astrocytes, with relevance for different mental disorders.

Reactive astrocytes synthesize GABA through the MAO-B enzyme, and a significant upregulation of MAO-B has been found in animal models of neurological disorders including Alzheimer’s, Parkinson’s and Amyotrophic Lateral Sclerosis [36,37,38], as well as during aging [39]. Gene-silencing of MAO-B or pretreatment with Selegiline, a selective and irreversible inhibitor, decreases astrocytic GABA-content and tonic GABA inhibition [22]. We confirmed that in our rat model of depression, Selegiline pretreatment of slices reversed the increase in tonic GABA inhibition. Moreover, pretreatment with Selegiline restored LTP, while not affecting synaptic inhibitory currents. Taken together, the effect of Selegiline and our initial observation that prefrontal slices from FSL rats showed a significant increase in LTP facilitation in the presence of the GABA_A_ receptor blocker revealed that plasticity in the prefrontal cortex was not directly impaired in this model of depression, but rather, that it was precluded by increased astrocyte-mediated inhibition. The fact that Selegiline did not have an effect on synaptic inhibitory currents shows that the attenuation of astrocyte-mediated tonic inhibition was sufficient to restore synaptic plasticity, and ruled out the role of neuronal inhibition. However, we are aware that Selegiline inhibition of MAO-B could potentially affect other transmission systems and signaling factors [40,41]. Particularly, the inhibition of MAO-B activity decreases the degradation of dopamine, a well-described bidirectional modulator of synaptic plasticity. The effect of dopamine on LTP is not completely understood; it has been shown that Dopamine D_1_ receptor agonists facilitate the maintenance of LTP [42], while activation of D_1_ and D_2_ receptors in combination rather facilitates the induction of LTD and inhibits LTP [43]. From this study, we cannot rule out the partial involvement of signals other than astrocytic GABA in the effect on synaptic plasticity.

Interestingly, Selegiline, also known as Deprenyl, has well characterized antidepressant effects and is used clinically as a transdermal treatment [44,45]. Again, the antidepressant effects have been attributed to increased levels of the monoamine system [46], but here, we provide an alternative astrocytic mechanism, where Selegiline restores the excitation/inhibition balance in the prefrontal cortex as well as synaptic plasticity by reducing astrocytic GABA synthesis. This effect is in line with current theories of depression being associated with an excitation/inhibition imbalance and/or a decrease in synaptic plasticity [47].

Several mechanisms have been shown to be affected in dysfunctional astrocytes that directly affect plasticity, and can mediate depressive-like behavior [48], including the glutamate/cysteine antiporter X_c_^−^ [49], reduced glutamate uptake [50], decreased perineuronal net formation [48] and reduced levels of D-serine [11]. Despite this, research on antidepressant treatments is typically neuron-oriented. The data presented here further emphasize the role of astrocytes in modulating neuronal activity and plasticity, and positions astrocytes as central targets in the search for novel and more efficient treatments for depression. To this end, we need to greatly enhance our understanding on astrocytic physiology and clearly clarify what a dysfunctional astrocyte is.

## Supplementary Materials

The following are available online at https://www.mdpi.com/2073-4409/9/7/1705/s1, Figure S1: In vitro Selegiline pretreatment doesn’t affect the amplitude or frequency of IPSCs in the prefrontal cortex of FSL rats.
