# Supplementary material for: Blocking Astrocytic GABA Restores Synaptic Plasticity in Prefrontal Cortex of Rat Model of Depression
Source: Cells. 2020 Jul 16;9(7):1705. doi: 10.3390/cells9071705 (PMC7408154; doi:10.3390/cells9071705)
Supplement: Supplementary file 1 [file cells-09-01705-s001.pdf]

Supplementary Figure

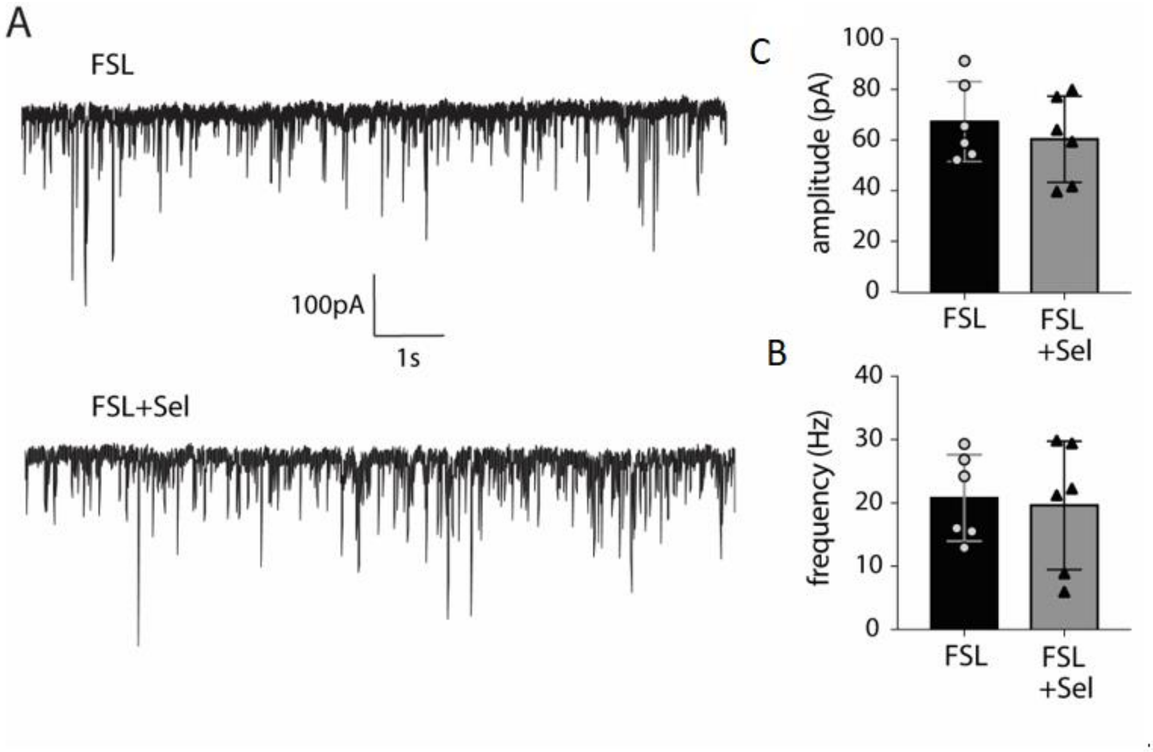

Figure S1. In vitro Selegiline pretreatment doesn't affect the amplitude or frequency of IPSCs in the prefrontal cortex of FSL rats. (**A**) Representative trace showing inhibitory postsynaptic current (IPSC) recordings from pyramidal neurons in prefrontal cortex (prelimbic area, layer five) in brain slices from FSL rats and FSL slices preincubated with Selegiline. No significant differences were observed between (**B**) mean IPSC frequency of FSL (20.8 ± 2.786) vs FSL with Selegiline (19.62 ± 4.133, *p*=0.81, t-test) and (**C**) mean IPSC amplitude of FSL (67.64 ± 6.444) vs FSL with Selegiline (60.69 ± 6.96, *p*=0.48, *t*-test). Bars indicate mean values, with SEM shown in error bars and the value of the individual recordings shown as symbols. FSL rats (*n*=6 from five animals) and FSL with Selegiline (*n*=6 from four animals).
